# Subcutaneous Estradiol Pellets as Hormone Therapy in Menopause: Clinical Pharmacology, Patient Selection and Safety Considerations

**DOI:** 10.3390/jcm15010048

**Published:** 2025-12-21

**Authors:** Leonardo Jacobsen, Daniela Maia Fernandes, Maria Luiza Nagel, Eline Lobo de Souza, Diogo Pinto da Costa Viana

**Affiliations:** 1 Brazilian Society for Research and Teaching in Medicine (SOBRAPEM), Sao Paulo 01318-901, Brazilpinto.viana@unifesp.br (D.P.d.C.V.); 2 Department of Gynecology, Escola Paulista de Medicina, Federal University of Sao Paulo (EPM-UNIFESP), Sao Paulo 04024-002, Brazil; 3 Brazilian Society of Obesity Medicine (SBEMO), Florianópolis 88070-800, Brazil

**Keywords:** estradiol, drug implants, estrogen replacement therapy, menopause, pharmacokinetics

## Abstract

**Background:** Among hormone therapy options for menopause, subcutaneous estradiol pellets offer sustained hormone release, avoid first-pass hepatic metabolism, and maintain a near-physiological estradiol-to-estrone ratio. Despite clinical use since the 1940s, standardized protocols remain lacking. **Methods:** We performed a critical narrative review following SANRA criteria. PubMed, Scopus, Embase, and LILACS were searched from 1949 to 2024 for randomized trials, cohort studies, and case series on estradiol pellets and outcomes in symptom control, bone health, pharmacokinetics, and safety. Animal studies, editorials, and reports without primary clinical data were excluded. **Results:** Following an initial peak within the first week, pellets maintain stable serum estradiol levels within the early-to-mid follicular range (50–113 pg/mL depending on dose) for four to six months, with a near-physiological E2:E1 ratio of approximately 1.5:1. The 25 mg dose achieves mean levels of 50–70 pg/mL, effectively controls vasomotor symptoms, and increases bone mineral density. Compared with oral estradiol, pellets bypass hepatic first-pass metabolism, resulting in neutral or favorable metabolic and thrombotic profiles. Compared with transdermal therapy, pellets provide more predictable pharmacokinetics, especially in women with low skin absorption. Safety concerns, including bleeding, tachyphylaxis, and supraphysiological levels, are mainly associated with excessive dosing, premature reimplantation, or lack of endometrial protection in women with a uterus. **Conclusions:** Estradiol pellets are an effective option for women with poor transdermal absorption, low adherence to daily regimens, or surgical menopause. Safety depends on clinical management with individualized dosing, adequate endometrial protection, and laboratory monitoring. Long-term comparative studies are needed to standardize protocols and support broader evidence-based use.

## 1. Introduction

Hormone therapy (HT) remains the most effective approach for the treatment of menopause-related symptoms, as well as for the prevention of its long-term consequences. Within the broad therapeutic arsenal available, the choice of the route of estrogen administration, which may be oral, transdermal, or subcutaneous, constitutes a crucial clinical decision that directly influences the pharmacokinetics, metabolic profile, efficacy, and safety of the treatment [1,2]. The individualization of therapy, therefore, constitutes a cornerstone of quality gynecological practice.

In this context, subcutaneous estradiol implants, commonly known as pellets, constitute one of the oldest and, paradoxically, most controversial routes of hormone replacement. Pellets, whose use has been documented since the 1940s [3,4], present both theoretical and practical pharmacokinetic advantages, particularly with regard to sustained hormone release and the avoidance of first-pass hepatic metabolism. This promotes more constant serum estradiol concentrations and a more physiological estradiol:estrone (E2:E1) ratio compared to oral administration [5,6].

Despite their long history and the existence of a commercially manufactured product in several countries until its global discontinuation for commercial reasons in 2011, pellet therapy lost ground to oral and transdermal formulations. However, a continued clinical demand for long-lasting therapy with high adherence ensured the availability of pellets through regulated compounding pharmacies in countries such as Brazil, the United States, and the United Kingdom. This growth in clinical practice, however, occurred in a context of fragmented knowledge and a lack of consistent guidelines, provoking a significant debate.

Current controversies do not result from failures associated with the subcutaneous route, but rather from poor clinical practice stemming from a lack of knowledge about its specific pharmacology. The use of excessive doses, inadequate intervals for reimplantation, and insufficient endometrial protection are examples of practices that resulted in adverse effects, such as supraphysiological estradiol levels, tachyphylaxis, and uterine bleeding [7,8].

This critical narrative review aims to synthesize the evidence on subcutaneous estradiol pellets as one option within menopausal hormone therapy. We analyze pharmacokinetic properties, clinical efficacy across multiple outcomes, and safety considerations when prescribed according to evidence-based protocols. Our objective is to provide clinicians with a factual, comprehensive assessment that clarifies appropriate patient selection, dosing strategies, and monitoring requirements.

## 2. Materials and Methods

A critical and narrative review of the literature was conducted to synthesize the evidence on the use of subcutaneous estradiol implants in female hormone therapy. The search strategy encompassed both historical investigations and contemporary studies, aiming to provide a comprehensive overview of the evolution of knowledge in the field. No commercial materials were directly used in this study; information regarding specific estradiol pellet products was reported as described in the original publications reviewed. No proprietary software requiring version identification was used beyond standard bibliographic database searches.

The methodological quality of this narrative review was evaluated using the SANRA scale (Scale for the Assessment of Narrative Review Articles), fulfilling all six recommended criteria for high-quality narrative reviews.

### 2.1. Data Sources and Search Strategy

A systematic search was carried out in the PubMed/MEDLINE, Scopus, Embase, and LILACS databases. This search was complemented by manual review of the reference lists of selected articles in order to identify additional relevant studies. The period considered included publications from January 1949 to September 2024.

Standardized medical subject headings (MeSH/DeCS) and free terms were used, combined with Boolean operators (AND, OR). The main strategy included expressions such as: (“Estradiol”[MeSH] OR estradiol[tiab]) AND (“Menopause”[MeSH] OR menopause[tiab] OR postmenopause[tiab]) AND (“Drug Implants”[MeSH] OR pellet*[tiab] OR “subcutaneous implant”[tiab] OR “estradiol implant”[tiab]).

### 2.2. Study Selection Criteria

Randomized clinical trials, cohort studies, case series, and reviews addressing estradiol pellets, either alone or with testosterone, in perimenopausal, postmenopausal women, or women with estrogen deficiency were included. The evaluated outcomes had to include clinical efficacy, pharmacokinetics, safety, or comparison with other routes. Studies with animals, opinion articles, editorials, and those without original data or whose primary focus was not estradiol were excluded.

### 2.3. Data Extraction and Analysis

The articles were individually analyzed regarding study type, sample, population, intervention, outcomes, results, and limitations. Data were organized by topic according to the sections of the present review, allowing a critical narrative analysis of the findings and controversies.

## 3. Results and Discussion

### 3.1. Pharmacokinetics of Estradiol Pellets

Subcutaneous estradiol implants function as long-acting reservoirs, releasing hormone directly into systemic circulation and bypassing first-pass hepatic metabolism [1,2].

**Figure 1:**
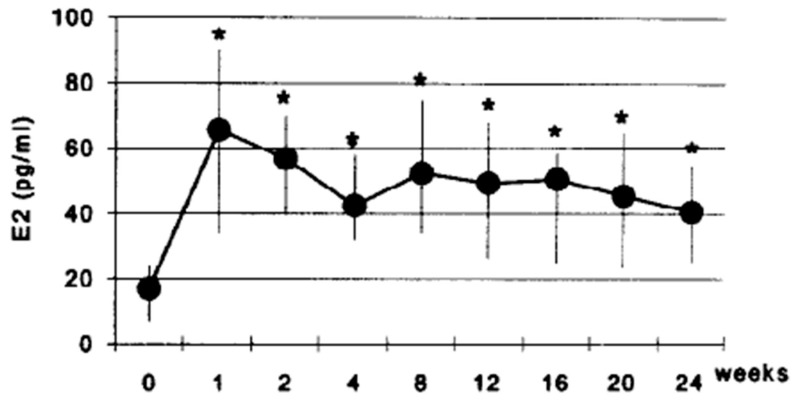
Serum estradiol concentrations (E2, pg/mL) over a 24-week period following subcutaneous implantation of estradiol pellets in postmenopausal women. The graph demonstrates a rapid rise in E2 levels within the first week, followed by a sustained plateau phase with relatively stable concentrations for up to six months. Error bars represent standard deviations. Asterisks indicate statistically significant differences from baseline (*p* < 0.05). Adapted from Cravioto et al. (2001) [10].

After subcutaneous insertion, crystalline estradiol pellets are slowly dissolved by interstitial fluid, providing gradual and continuous absorption. A study with a 25 mg pellet demonstrated maintenance of serum estradiol levels between 50 and 70 pg/mL for three months, with a progressive decline still above baseline values for up to six months [5]. In a randomized clinical trial, administration of two 25 mg pellets (total of 50 mg) resulted in mean levels of 113 pg/mL in the first week, which remained stable over 24 weeks [9]. Pharmacokinetic studies with the commercial implant Riselle^®^ 25 mg demonstrate gradual absorption reaching peak concentration of approximately 68 pg/mL within 10 h, without an abrupt burst effect. Levels at week 1 average 66 pg/mL, followed by gradual decline to a sustained plateau. As presented in Figure 1, mean estradiol levels remained stable between 40 and 60 pg/mL from week 2 throughout the 24-week observation period [10].

For physiological context, estradiol levels in premenopausal women range from 30–120 pg/mL during the early follicular phase to 80–400 pg/mL in the mid-follicular and ovulatory phases. The optimal plasma estradiol concentration for hormone therapy is 60–150 pg/mL: levels of 60 pg/mL relieve hot flashes in 50% of women and prevent bone resorption, while levels approaching 100 pg/mL achieve complete symptom relief and bone accretion in most women [11]. The levels observed with 25 mg pellets (40–70 pg/mL) fall within the early follicular range and reach the threshold for therapeutic efficacy.

It is important to note considerable inter-individual variability in serum levels, with standard deviations ranging from ±16 to ±37 pg/mL at different timepoints, as illustrated in Figure 1 [10]. This indicates that some women achieve levels considerably above or below the mean despite identical dosing, underscoring the importance of individualized monitoring rather than fixed protocols.

The dose is the main determinant of the levels achieved, although there is interindividual variation. The 25 mg dose, frequently used at the start of treatment, raises levels to the follicular phase range. Studies report medians of 103 pg/mL after two weeks and 87 pg/mL after one year of use [12,13]. Higher doses, such as 50 or 100 mg, produce higher concentrations [14]. Mean levels of 94 pg/mL with 50 mg and 164 pg/mL with 100 mg have been described [6]. Regarding whether two 25 mg pellets produce the same pharmacokinetic profile as a single 50 mg pellet, available data suggest approximate dose proportionality, with 113 pg/mL achieved with two 25 mg pellets [9] compared to 94 pg/mL with a single 50 mg pellet [6]. However, direct comparative trials are lacking, and it remains unclear whether surface-area-to-volume differences between multiple smaller pellets and a single larger pellet might alter dissolution kinetics and result in distinct absorption patterns.

An additional benefit is the maintenance of a physiological E2:E1 ratio, generally between 1.5:1 and 2:1, with predominance of circulating estradiol. This contrasts with the oral route, where estrone predominates, and brings the hormonal profile closer to the premenopausal pattern [5,6,10]. This distinction is clinically relevant because estradiol binds with higher affinity to estrogen receptors α and β than estrone, producing more potent estrogenic effects and potentially achieving therapeutic benefits at lower total estrogen exposure.

The duration of the effect is prolonged and often exceeds the standard six-month reimplantation interval. Elevated levels were observed for more than 15 months with doses of 50 to 75 mg [15]. In a clinical audit, the median time to return to baseline levels after a 50 mg implant was 10.2 months [16]. This long duration has important clinical implications, especially for the planning of reimplantation and continuous endometrial protection.

### 3.2. Evidence of Clinical Efficacy

The assessment of the efficacy of any modality of hormone therapy must consider multiple domains that influence the quality of life of women during menopause. The literature on subcutaneous estradiol implants, although composed of studies with different methodological designs and levels of evidence, offers consistent data on relevant clinical outcomes. In this section, the available evidence regarding the impact of estradiol pellets on bone health, the control of vasomotor and psychological symptoms, sexual function, and body composition will be analyzed. To provide a summary of the evidence, Table 1 presents the main studies that investigated these outcomes.

### 3.3. Bone Health

The maintenance of bone mass and the prevention of osteoporosis are central indications of hormone therapy in menopause. In this domain, the scientific literature presents solid evidence regarding the high efficacy of subcutaneous estradiol implants. Unlike therapies focused only on slowing bone loss, studies with pellets demonstrate significant and lasting increases in bone mineral density (BMD).

This effect is evident even with low doses. The use of 25 mg pellets for one year resulted in median increases of 5.65% in lumbar BMD and 3.36% in total hip BMD, surpassing the loss observed in the control group [13]. Similarly, there was a 5.6% gain in lumbar BMD with the same dose associated with continuous progestin [30].

The bone response is proportional to the serum levels achieved. An RCT demonstrated a positive correlation between estradiol levels and BMD gain; women who maintained levels above 82 pg/mL did not show bone loss in clinically relevant sites [22].

The positive effect is also sustained in the long term. In a three-year longitudinal study, an average annual gain of 3.3% in spinal trabecular BMD was observed [21]. A cross-sectional study with users of low-dose pellets for approximately 16 years revealed BMD 20% to 25% higher than age-matched controls, challenging the idea that bone loss is inevitable with aging [20].

The role of adjuvant testosterone is still debated. One study showed a faster and more intense BMD increase with the combination of estradiol and testosterone (Davis et al., 1995) [23], but others did not confirm this additional benefit, suggesting that estradiol is primarily responsible for the protective effect [19,27].

The magnitude of the gains, especially with high doses and prolonged use, indicates a possible action beyond simple inhibition of bone resorption. Histomorphometric studies have demonstrated increased trabecular thickness in users of high-dose pellets, indicating greater osteoblastic activity and suggesting a potential direct anabolic effect on bone [25,26].

### 3.4. Vasomotor Symptoms and Psychological Well-Being

The control of vasomotor symptoms (VMS) is the main indication for hormone therapy in menopause, and estradiol pellets demonstrate high efficacy in this context. Studies show rapid and complete relief of hot flashes, with improvement already within the first 48 h after implantation [5,15].

The minimum effective dose was investigated in a randomized, double-blind clinical trial that compared 25 mg and 50 mg of estradiol in women with surgical menopause. Although the 50 mg dose produced higher serum levels, there was no significant difference in symptom relief regarding intensity or duration; both provided control for approximately 5.6 to 5.9 months, supporting the 25 mg dose as the most appropriate initial choice [14].

In addition to VMS, pellets positively impact psychological well-being. Several studies report significant improvement in symptoms such as anxiety, irritability, and depressive mood. In an RCT, the use of estradiol, either alone or with testosterone, was superior to placebo in reducing depression and anxiety scores after two months. Although a placebo effect was observed, the onset of response was faster in the treated group, which is clinically relevant in the management of patients with affective symptoms [18]. The hormonal stability provided by the implants, by avoiding daily fluctuations in serum levels, may be a determining factor in this benefit [2].

Efficacy in refractory patients also deserves attention. In an observational study with women followed in a specialized clinic, the main reason for using pellets was therapeutic failure with other forms of MHT. Even in a group predominantly composed of complex cases, the implants showed a high degree of satisfaction and satisfactory symptom relief, especially regarding emotional symptoms, positioning themselves as a valid alternative when other routes fail [31].

### 3.5. Sexual Function and Body Composition

Hormonal implants demonstrate relevant positive effects on sexual function and body composition, fundamental aspects for quality of life and metabolism in postmenopause.

Decreased libido and sexual dysfunction are common climacteric complaints. Estradiol alone improves indirect contributors such as dyspareunia and urogenital atrophy through tissue restoration, though its direct effect on libido is modest. Although some studies combined estradiol with testosterone to address hypoactive sexual desire disorder [23,32], the discussion of androgen supplementation remains controversial and is beyond the specific scope of this review on estradiol safety and efficacy.

Regarding body composition, the available data are favorable and contradict the perception that hormone therapy leads to weight gain. In a controlled study, women treated with pellets containing 20 mg of estradiol did not show weight gain or an increase in body fat percentage compared to the control group [28]. More importantly, an 18% reduction in the abdominal fat/lean mass ratio was observed, indicating favorable fat redistribution and potential benefit in reducing cardiometabolic risk.

### 3.6. Safety Analysis and Adverse Effects

The safety profile of estradiol implants is intrinsically linked to their pharmacological properties. Reported adverse outcomes, including endometrial hyperplasia, tachyphylaxis, and supraphysiological estradiol levels, correlate with specific dosing patterns and monitoring practices rather than inherent risks of the subcutaneous route.

In this regard, it is essential to recognize that estradiol pellets deliver 17β-estradiol, identical to the active ingredient in approved transdermal and oral bioidentical formulations. Therefore, the absolute contraindications do not differ from the general guidelines established by major organizations such as the North American Menopause Society (NAMS) and the International Menopause Society (IMS). These include undiagnosed abnormal genital bleeding, known or suspected breast cancer, active deep vein thrombosis (DVT) or pulmonary embolism, and active liver disease [33,34]. Similarly, the adverse event profile is consistent with the known systemic effects of estrogen. Common side effects reported in clinical practice include breast tenderness (mastalgia), fluid retention, bloating, nausea, and headaches or migraines. These effects are typically dose-dependent [8,14].

Among the main challenges are adequate endometrial protection, the management of tachyphylaxis and supraphysiological estradiol levels, the control of cardiovascular risk, and the properties of compounded formulations. To a large extent, these factors are preventable and subject to strict control. This section will address the central safety aspects associated with estradiol implants, analyzing pharmacological foundations, dosing criteria, and monitoring recommendations in light of the available literature.

### 3.7. Endometrial Safety

Endometrial protection is one of the pillars in the management of estradiol therapy in women with a uterus. As with any form of systemic estrogen, the isolated use of estradiol pellets stimulates endometrial proliferation, substantially increasing the risk of irregular uterine bleeding, hyperplasia, and, in the long term, endometrial carcinoma. This risk has been documented since the first reports on hormonal implants, with historical series describing bleeding rates between 60% and 100% in women without hormonal opposition [3,4]. In a later RCT, the use of a 25 mg pellet without opposition led to bleeding in 75% of patients, of whom 42% required hysterectomy [29].

The association of a progestogen is therefore mandatory. The choice of molecule and administration regimen must ensure both effective histological protection and a tolerable bleeding pattern. Although oral micronized progesterone is a valid option, synthetic progestins are often preferred due to their greater potency and receptor affinity, offering more consistent endometrial protection [35].

The attempt to maintain a continuous, bleeding-free regimen by combining estradiol pellets with daily oral progestins presents relevant challenges in clinical practice. In a prospective study, the attempt to maintain amenorrhea with daily oral progestin resulted in an irregular menstrual pattern and intense or continuous breakthrough bleeding, leading to treatment discontinuation in more than 70% of patients. Although biopsies showed atrophy, the bleeding pattern was considered clinically unacceptable, probably due to a pharmacokinetic mismatch between the constant release of estradiol from the pellet and the fixed administration of oral progestin [36].

More modern approaches have shown superior results. The combination of a 25 mg estradiol pellet with continuous norethisterone (1 mg/day) achieved amenorrhea in 63% of users after one year, with no cases of hyperplasia [30]. An even more effective strategy is the association of pellets with a levonorgestrel-releasing intrauterine device (LNG-IUS), which resulted in amenorrhea in 72% of women and complete endometrial protection over 12 months [37]. Given its superior efficacy and excellent adherence profile, this combination has been considered by many authors as the preferred option in clinical practice.

### 3.8. Tachyphylaxis and the Risk of Supraphysiological Levels

Tachyphylaxis is a clinically relevant phenomenon in the use of estradiol implants, characterized by the early return of climacteric symptoms and the request for new implants by the patient, even in the presence of serum estradiol levels still within the therapeutic range or even elevated [7,38]. This dissociation between symptoms and hormonal dosages represents a significant challenge in clinical practice.

Classic reports had already documented this paradox in women using doses equal to or greater than 50 mg, presenting symptoms compatible with estrogen deficiency despite estradiol concentrations above 327 pg/mL [7]. Even so, markedly supraphysiological levels, above 477 pg/mL, were infrequent, with a prevalence estimated at about 3% in large cohorts [39]. Tachyphylaxis is believed to have a multifactorial origin. One hypothesis proposes that the recurrence of symptoms does not result from low absolute levels but from an abrupt decline after a high peak, generating a condition similar to “hormonal withdrawal.” Another possible explanation was demonstrated in a clinical trial with chronic implant users, in which reimplantation with placebo promoted symptom improvement comparable to the active pellet, suggesting a non-hormonal component involved [24].

The main risk associated with tachyphylaxis is progressive hormonal accumulation, especially when new implants are inserted based solely on symptomatology. In this situation, serum levels may remain chronically elevated, with potential clinical implications [40]. This pattern appears to be related to the use of doses starting at 50 mg. In contrast, studies with lower doses, such as that of Owen et al. [12], did not observe this issue, suggesting that the lower variability and more physiological levels associated with the 25 mg dose may reduce the risk of tachyphylaxis.

Safe clinical management requires prudence. It is recommended that new implants not be inserted while serum estradiol levels remain elevated [41]. Safe alternatives include delaying reimplantation, reducing the subsequent dose, or temporarily using short-acting formulations such as transdermal gel for symptom control until hormonal normalization. Monitoring serum levels, therefore, is an essential tool to guide clinical decisions and minimize the risk of overdose.

### 3.9. Cardiovascular Safety and Thrombotic Risk

Cardiovascular risk, especially venous thromboembolism (VTE), is one of the main safety criteria in hormone therapy. The route of estrogen administration is the primary determinant of this risk.

Oral therapies increase the risk of VTE by up to four times due to first-pass hepatic metabolism, which stimulates the production of coagulation factors and reduces sensitivity to activated protein C [1,2]. In contrast, parenteral routes such as transdermal bypass this metabolism and do not significantly increase thrombotic risk, as demonstrated in large observational studies such as the ESTHER study [42].

Although there are no clinical trials specifically designed to assess the incidence of VTE with the use of subcutaneous estradiol implants, it is plausible to assume, based on the pharmacological mechanism, that pellets share the same safety profile as the transdermal route. Both avoid hepatic stimulation associated with increased thrombotic risk. This hypothesis is supported by studies that analyzed intermediate outcomes; for example, the use of pellets in doses of 25 to 50 mg did not promote negative changes in coagulation parameters [4,43].

Moreover, data on the lipid profile reinforce the metabolic safety of pellets. Unlike the oral route, which is frequently associated with increased triglycerides, implants have shown a neutral or even favorable effect, with a trend toward reduced triglycerides, increased HDL cholesterol, and decreased LDL cholesterol [5,17].

Therefore, considering the mechanisms involved and the available data on surrogate markers, estradiol pellets present a cardiovascular safety profile comparable to that of the transdermal route, being a preferable option over the oral route, especially in women at elevated thrombotic risk.

### 3.10. Compounded Formulations and the Importance of Standardization

The commercial availability of estradiol pellets, such as Riselle^®^ (Organon), ceased globally around 2011. It is crucial to note that this withdrawal was primarily driven by strategic commercial decisions rather than safety or efficacy concerns. Consequently, clinical practice in many regions now relies on compounded formulations. This scenario has prompted debates about the safety and consistency of these preparations.

Two retrospective studies illustrate this relationship well. Jiang et al. [8], in a cohort based on medical records from a single institution, compared users of compounded pellets with users of FDA-approved therapies. The results indicated a higher incidence of adverse effects and supraphysiological levels in the pellet group. The analysis suggests that the outcome is related to overdosing, since the mean estradiol levels in this group reached 238 pg/mL, a value substantially higher than the 80–100 pg/mL generally observed with 25 mg implants, which are considered effective and safe [10,13].

In contrast, Donovitz [44] analyzed a database with more than one million implants performed by physicians trained in a standardized protocol. The study reported a complication rate below 1%, suggesting that the adoption of a structured method, with dose control and rigorous follow-up, may confer a high degree of safety. Despite methodological limitations, such as reporting bias and potential conflict of interest, the data point to the importance of the care model over the formulation type.

The comparison between studies highlights that the determining factor for safety is not the pharmacy but the prescriber’s conduct. Therapeutic individualization, the use of physiological doses, laboratory monitoring, and the application of well-defined protocols are essential to mitigate risks and optimize benefits. This scenario reinforces the need for continued medical education and clear clinical guidelines to guide the responsible use of compounded pellets.

### 3.11. Therapeutic Comparisons with Other Routes of Estradiol Administration

The route of estrogen administration is one of the most relevant decisions in the individualization of hormone therapy. Each route has distinct pharmacokinetic properties, metabolic effects, and safety profiles, requiring careful analysis according to the clinical goals and risk factors of each patient. This section presents a comparison between the subcutaneous route via pellets and the oral and transdermal routes, widely used in medical practice.

### 3.12. Implants Versus Oral Therapy

The main difference between pellets and the oral route lies in first-pass metabolism. Although oral estradiol is well absorbed, it undergoes extensive metabolism in the intestine and liver before reaching systemic circulation, which reduces its bioavailability to only 2% to 10% and promotes predominant conversion to estrone and its conjugates, such as estrone sulfate [45]. This results in a non-physiological hormonal profile, with an E1:E2 ratio that may exceed 5:1, in contrast to the premenopausal pattern, in which estradiol predominates [2].

Subcutaneous implants avoid this hepatic metabolism, releasing estradiol directly into systemic circulation. As a result, they allow the use of lower doses, achieve stable therapeutic levels, and maintain an E2:E1 ratio greater than 1.0, closer to the physiological hormonal environment [5,6].

This metabolic difference has a direct impact on the safety profile. The oral route increases triglycerides, c-reactive protein, and coagulation factors, raising thrombotic risk. Pellets, by avoiding hepatic stimulation, share with the transdermal route a safer profile in these parameters [1,45].

Another advantage of implants is their neutrality on sex hormone-binding globulin (SHBG). Oral estradiol significantly increases SHBG, which reduces free and bioavailable testosterone, potentially affecting libido, energy, and well-being. The subcutaneous route does not induce this increase, preserving endogenous testosterone levels and offering better clinical performance in women with sexual complaints [1].

The main limitation of pellets is reduced therapeutic flexibility. While the oral route allows rapid dose adjustments or immediate discontinuation, implants require planning, as they cannot be easily removed after insertion. Dose adjustment occurs only in the next cycle, which demands rigorous follow-up.

### 3.13. Implants Versus Transdermal Therapy

The comparison between the subcutaneous route via pellets and the transdermal route via patches or gels requires a more in-depth analysis, since both are parenteral routes that avoid first-pass hepatic metabolism. For this reason, they share a similar and favorable safety profile in terms of thrombotic risk and adverse metabolic effects [2]. The main distinctions between these approaches lie in the pharmacokinetics of hormone release, absorption variability, and practical consequences for treatment adherence.

Several factors, such as individual skin permeability, degree of local adiposity, cutaneous blood flow, temperature, and application technique, can generate considerable variations in serum estradiol levels among women using the same product at the same dose [46,47]. In a crossover study, Järvinen et al. [48] documented high inter- and intraindividual variability, with coefficients of variation in estradiol exposure of up to 35% for gel and 39% for patch. This resulted in differences of up to 11-fold in maximum concentration among gel users and up to 7-fold with patches. These data confirm that transdermal absorption is inherently unstable, even with industrially manufactured products. Predictability of response is not guaranteed solely by the pharmaceutical origin of the product.

This variability implies that many women, even with correct use, do not reach therapeutic levels. According to Kuhl [2], up to 30% of users of a 50 µg/day patch remain at subtherapeutic levels. In a more recent study, Glynne et al. [11] demonstrated that 25% of women using the highest transdermal doses still presented estradiol below 54 pg/mL, the minimum threshold for bone protection. This confirms the existence of a subgroup with deficient cutaneous absorption, the so-called “poor absorbers.”

This clinical scenario gives subcutaneous implants a particularly relevant therapeutic role. For women with low transdermal absorption or fluctuating symptoms despite proper use of gel or patch, implants are not only an alternative but often the only effective and stable route of administration. Since they are absorbed directly from subcutaneous tissue, pellets eliminate the uncertainties imposed by the cutaneous barrier. Clinical trials have already demonstrated superior stability in serum estradiol levels with the use of implants for up to 24 weeks [9].

The International Menopause Society (IMS) acknowledges this issue. In its most recent White Paper, it states that “a transdermal preparation may not always be the best option for an individual” [33]. As an alternative, it suggests the oral route, which leaves women with contraindications to oral administration, such as thrombotic risk or hypertriglyceridemia, without adequate options. In this context, pellets emerge as the most predictable and safest parenteral route.

In summary, the transdermal route offers flexibility and reversibility, but with unpredictable absorption for some patients. Subcutaneous implants, in turn, provide greater pharmacokinetic predictability, with stable hormone delivery and full adherence for several months, although with less flexibility and the need for a procedure for insertion. The main differences between administration routes are summarized in Table 2.

### 3.14. Recommendations for Clinical Practice

The safe and effective incorporation of estradiol pellets into menopause management requires therapeutic individualization, mastery of pharmacokinetic principles, and appropriate clinical-laboratory monitoring. Based on the reviewed literature, it is possible to establish objective recommendations to assist the prescriber in the responsible and evidence-based use of this therapeutic modality.

#### Patient Selection

The application of scientific evidence to clinical practice must be patient-centered. Given the variety of options for estrogen therapy, each with a distinct profile of benefits and limitations, there is no universal approach. As highlighted by Laing and Hillard [38], “a clinician’s role is to guide and help women make evidence based, unbiased and informed choices.” The same perspective appears in the guidelines of the North American Menopause Society, which in its 2022 guidelines states that “in the absence of contraindications, a woman should determine her preferred hormone therapy formulation, dose, and duration of use, with ongoing assessment and shared decision-making with her healthcare professional” [34].

However, the choice of this route requires careful consideration of its intrinsic procedural and pharmacokinetic characteristics. Unlike non-invasive methods, pellet insertion requires a minor surgical procedure, which represents a barrier for some patients [1]. Furthermore, the prolonged duration of action, while beneficial for adherence, implies a lack of immediate reversibility. As highlighted by Wheatley et al. [16], this makes patient selection critical, as the hormonal release cannot be abruptly terminated in the event of adverse effects or intolerance, unlike oral or transdermal formulations. Consequently, clinical prudence suggests prioritization of this modality for specific clinical scenarios where these unique properties offer a distinct advantage.

Although pellets may be used by any woman with an indication for hormone therapy, they become especially valuable in specific contexts. Their main indication is in cases of refractory symptoms or therapeutic failure with conventional routes [31], including women with low cutaneous absorption, in whom subcutaneous administration ensures adequate estradiol levels not achieved by gels or patches [11,48]. Another relevant indication includes patients with low adherence to daily or weekly regimens, as pellets offer continuous and sustained release. They also stand out as an effective and practical option for women in surgical menopause or with premature ovarian insufficiency, who often require sustained doses for cardiovascular and bone protection.

### 3.15. Dosage and Reimplantation Interval

The choice of dose and reimplantation interval represents one of the most critical aspects to ensure safety and efficacy in estradiol pellet therapy. The reviewed literature identifies the 25 mg dose as the preferred standard for most patients. This dosage achieves therapeutic and physiological serum levels, with proven efficacy in relieving vasomotor symptoms, improving psychological well-being, and protecting bone health [10,12,13].

Randomized clinical trials show that although higher doses such as 50 mg promote higher serum levels, there is no clinical superiority in symptom control compared to the 25 mg dose [14]. For this reason, the routine use of higher doses, such as 50 mg or 100 mg, should be considered outdated and not recommended when the goal is symptomatic relief. In addition to offering no additional benefits, these doses increase the risk of dose-dependent adverse effects such as mastalgia, irregular uterine bleeding, tachyphylaxis, and hormonal accumulation.

The interval between reimplantations should not be fixed, as the duration of pellet action may exceed six to ten months in many patients [15,16]. The practice of performing reimplantations at regular intervals without careful evaluation is one of the main causes of hormonal accumulation and the development of tachyphylaxis. The clinical decision should follow a two-step strategy: first, the presence of persistent climacteric symptoms suggesting depletion of the previous implant; second, laboratory confirmation that serum estradiol levels have returned to baseline or low values. This dual verification is essential to avoid premature reimplantations, minimize the risk of hormonal accumulation, and protect the patient from prolonged exposure to supraphysiological concentrations, being indispensable for a safe, effective, and best-practice-aligned approach to the use of subcutaneous implants.

### 3.16. Monitoring and Patient Education

The success of estradiol pellet therapy depends on structured monitoring and effective communication between physician and patient. Clinical follow-up should be regular, allowing continuous evaluation of symptomatic response and early detection of adverse effects such as mastalgia or, in women with a uterus, any pattern of abnormal bleeding that requires appropriate investigation to exclude endometrial pathology.

In addition, patient education and informed consent are indispensable steps in the responsible management of this therapeutic modality. The woman must receive clear guidance on the characteristics of the subcutaneous route, understanding both its benefits and its limitations, including the impossibility of immediate discontinuation after insertion. It is essential that expectations are well aligned from the beginning, that there is understanding about the need for continuous endometrial protection in indicated cases, and that the patient is able to recognize signs and symptoms that require medical evaluation. A well-informed patient actively participates in the shared decision-making process, which constitutes the foundation for safe, ethical, and effective hormone therapy.

## 4. Conclusions

Subcutaneous estradiol implant therapy, despite its long historical trajectory, remains the subject of clinical and regulatory debate. This critical review, by gathering evidence from both classical and contemporary studies, provides consistent foundations to guide current medical practice.

Pellets present stable pharmacokinetics, with prolonged release and more physiological metabolism by avoiding hepatic passage. This translates into high efficacy in the control of vasomotor and psychological symptoms, as well as sustained benefits for bone health, with indications of anabolic action. The 25 mg dose proves to be the most appropriate for initiating treatment, with an excellent efficacy and safety profile.

Safety concerns generally do not arise from the subcutaneous route itself but from failures in clinical conduct. Risks such as endometrial hyperplasia, tachyphylaxis, and supraphysiological levels are preventable with adequate endometrial protection, laboratory monitoring, and careful individualization of the dose. Programmed reimplantations at fixed intervals, without clinical and biochemical evaluation, represent one of the main causes of avoidable adverse events. The controversy, therefore, reflects more a gap in the prescriber’s technical training than intrinsic problems with the route.

Pellets should be considered a strategic option within the therapeutic arsenal, especially for women with poor transdermal absorption, low adherence to conventional regimens, or early surgical menopause. Their safe and effective use requires clear clinical guidelines and continued medical education based on evidence.

Finally, gaps remain that require investigation. There is a lack of randomized clinical trials with larger samples and long-term follow-up comparing pellets to other routes regarding relevant clinical outcomes. Studies on bioequivalence, consistency, and quality control of compounded formulations are also essential, especially after the discontinuation of industrialized products, to ensure safe and science-based access for all women who may benefit from the therapy.

## Figures and Tables

**Table 1:** Summary of Studies on Estradiol Pellets.

Author (Year)	Study Design	Population (N)	Intervention vs. Comparison	Duration	Main Outcome(s)	Main Conclusion
Lobo et al. (1980) [5]	Prospective controlled study	22 post-hysterectomy vs. 20 controls	E2 25 mg implant vs. no treatment	6 months	Hormone levels, Lipid profile, Hot flashes	25 mg implant is effective for symptom control and HDL improvement over 6 months
Thom et al. (1981) [6]	Observational study	24 post-hysterectomy	E2 100 mg vs. 50 mg vs. 50 mg + T 100 mg vs. T 200 mg	Up to 12 months	Hormone profiles	E2 implants (50 and 100 mg) achieve physiological levels and suppress FSH
Kapetanakis et al. (1982) [15]	Case series	10 with estrogen deficiency	E2 (25–75 mg) ± T (75 mg)	Up to 76 weeks	Hormone levels, Symptom relief	E2 ≥ 250 mg effective for symptom relief and long-term E2 maintenance (>70 weeks)
Sharf et al. (1985) [17]	Observational study	8 post-hysterectomy	E2 100 mg vs. baseline	6 months	Lipid profile	Improves HDL, reduces LDL, no effect on triglycerides
Montgomery et al. (1987) [18]	RCT, Double-blind, Placebo-controlled	84 peri/postmenopausal	E2, E2 + T pellets vs. placebo	2–4 months	Psychological symptoms	Superior to placebo at 2 months; equal at 4 months due to placebo effect
Stanczyk et al. (1988) [9]	RCT	20 postmenopausal, hysterectomized	E2 pellets (2 × 25 mg) vs. patch (0.1 mg)	24 weeks	Pharmacokinetics, Lipids, Bone markers	Pellets provide more stable estradiol levels than patch
Owen et al. (1992) [12]	Observational study	12 postmenopausal	E2 25 mg vs. baseline	28–35 weeks	Symptoms, Estradiol levels	25 mg effective >6 months; should be first choice
Garnett et al. (1992) [19]	RCT with control	50 postmenopausal + 25 controls	E2 75 mg vs. E2 75 mg + T 100 mg	1 year	BMD	T does not add benefit; effect depends on E2 level
Naessén et al. (1993) [20]	Controlled cross-sectional	70 (35 treated vs. 35 control)	E2 20 mg long-term vs. control	Mean 16 years	BMD (various sites)	Low-dose implants preserve bone with age
Holland et al. (1994) [13]	Controlled clinical trial	36 postmenopausal (18 vs. 18)	E2 25 mg vs. no treatment	1 year	BMD (spine and hip)	E2 25 mg increases BMD and prevents bone loss
Ryde et al. (1994) [21]	Longitudinal study	29 postmenopausal, hysterectomized	E2 25–100 mg	3 years	BMD, Body calcium	Significant, sustained BMD increase over 3 years
Studd et al. (1994) [22]	RCT with control	45 postmenopausal vs. 15 controls	E2 pellets (25, 50, 75 mg) vs. none	1 year	BMD, E2 levels	BMD gain is E2 dose-dependent; >82 pg/mL prevents loss
Davis et al. (1995) [23]	RCT, Single-blind	34 postmenopausal	E2 50 mg vs. E2 50 mg + T 50 mg	2 years	BMD, Sexual function, Lipids	T addition enhances BMD and sexual function
Pearce et al. (1997) [24]	RCT, Double-blind, Placebo-controlled	40 chronic users	E2 50 mg reimplant vs. placebo	2 months	Psychological symptoms	No difference from placebo; symptoms not hormone-related
Wahab et al. (1997) [25]	Controlled pilot study	24 post-hysterectomy (12 vs. 12)	E2 implant vs. none	≥15 years	BMD (spine and hip)	Long-term implants preserve BMD better than expected
Vedi et al. (1999) [26]	Case–control study	24 (12 implants vs. 12 controls)	Long-term E2 implant vs. premenopausal	≥14 years	Bone histomorphometry	Shows anabolic effect and increased formation
Panay et al. (2000) [14]	RCT, Double-blind	44 surgical menopause	E2 25 mg vs. E2 50 mg	6 months	Climacteric symptoms	25 mg is as effective as 50 mg
Sands et al. (2000) [27]	Longitudinal study	25 surgical menopause	E2 50 mg vs. E2 50 mg + T 100 mg	32 weeks	Bone turnover markers	T increases bone formation
Cravioto et al. (2001) [10]	Open observational	15 postmenopausal, hysterectomized	E2 25 mg vs. baseline	24 weeks	Hormones, Symptoms, Metabolism	25 mg improves symptoms and hormones without metabolic harm
Hansen et al. (2003) [28]	Controlled cohort	34 postmenopausal (20 vs. 14)	E2 20 mg vs. control	64 weeks	BMD, Fat distribution	E2 improved BMD and reduced abdominal fat
Rufford et al. (2003) [29]	RCT, Double-blind, Placebo-controlled	40 with urgency syndrome	E2 25 mg vs. placebo	6 months	Urinary symptoms, Endometrial safety	No benefit; high bleeding and hysterectomy risk
Wheatley et al. (2016) [16]	Retrospective audit	114 postmenopausal	E2 50 mg (Estrapel)	~311 days	Pharmacokinetics, Adverse events	Single implant lasts >12 months; most common AE: vaginal bleeding
Jiang et al. (2021) [8]	Retrospective cohort	539 postmenopausal (384 pellets vs. 155 FDA HT)	Pellets vs. FDA-approved HT	Mean 3.9 years	Side effects, Hormone levels (E2, T)	Pellets have higher adverse effects and supraphysiological hormone levels

List of abbreviations: RCT = Randomized Clinical Trial; N = Number of participants; E2 = Estradiol; T = Testosterone; BMD = Bone Mineral Density

**Table 2:** Comparison of the Main Routes of Estradiol Administration in Hormone Therapy.

Parameter	Oral Route	Transdermal Route (Patch/Gel)	Subcutaneous Route (Pellet)
First-Pass Hepatic Metabolism	Extensive first-pass metabolism to estrone and conjugates	Bypasses liver; absorbed through skin	Bypasses liver; direct to bloodstream
Estradiol:Estrone Ratio (E2:E1)	Non-physiological (≈1:5)	Physiological (≈1:1)	Physiological (1.5:1)
Serum Levels	Fluctuating (peaks and valleys)	Variable (irregular absorption)	Stable and sustained
VTE Risk	Increased (2–4× VTE risk)	Not increased	Not increased (theoretical)
Effect on Triglycerides	Increases triglycerides	Neutral effect	Lowers triglycerides
Impact on SHBG	Significantly increases	Minimal/neutral effect	Minimal/neutral effect
Treatment Adherence	Requires daily dosing	Requires daily or weekly application	Guaranteed for months
Therapy Flexibility	Easy adjustment/discontinuation	Easy adjustment/discontinuation	Removal not possible
Management of Uterine Bleeding	Usually responsive to progestagen adjustment; easy discontinuation if needed	Managed by dose adjustment or switching preparation; allows immediate discontinuation	LNG-IUS is the preferred option; with adjustment of the progestagen regimen as an alternative.

List of abbreviations: VTE = Venous Thromboembolism; SHBG = Sex Hormone-Binding Globulin; LNG-IUS = Levonorgestrel-releasing Intrauterine Device.

## Data Availability

Not applicable. No original datasets were generated or analyzed in this study.

## Abbreviations

The following abbreviations are used in this manuscript:

BMD	Bone Mineral Density
DeCS	Descritores em Ciências da Saúde
E1	Estrone
E2	Estradiol
FDA	Food and Drug Administration
FSH	Follicle-Stimulating Hormone
HDL	High-Density Lipoprotein
HT	Hormone therapy
IMS	International Menopause Society
LDL	Low-Density Lipoprotein
LILACS	Literatura Latino-Americana e do Caribe em Ciências da Saúde
LNG-IUS	Levonorgestrel-Releasing Intrauterine System
MEDLINE	Medical Literature Analysis and Retrieval System Online
MeSH	Medical Subject Headings
N	Number of participants
RCT	Randomized Clinical Trial
SANRA	Scale for the Assessment of Narrative Review Articles
SHBG	Sex Hormone-Binding Globulin
T	Testosterone
TIAB	Title/Abstract
VTE	Venous Thromboembolism
